# Free‐Breathing 3D Whole Heart and Aorta Cine MRI Without Contrast Agent—Comparison to Clinical Standard

**DOI:** 10.1002/jmri.70343

**Published:** 2026-04-20

**Authors:** Ruixin Chen, Huangling Lu, Alexandru Cernicanu, Jos Westenberg, Merlijn Sevenster, Jochen Keupp, Jakob Meineke, Hildo J. Lamb

**Affiliations:** ^1^ Department of Radiology, Cardio Vascular Imaging Group (CVIG) Leiden University Medical Centre Leiden the Netherlands; ^2^ Department of Radiology Amsterdam University Medical Centre Amsterdam the Netherlands; ^3^ Philips Benelux Eindhoven the Netherlands; ^4^ Philips Research Hamburg Germany

**Keywords:** 3D cine, cartesian spiral sampling, free‐breathing MRI, quantitative cardiac MRI

## Abstract

**Background:**

The demand for cardiac MRI is increasing with the growing burden of cardiovascular disease. However, conventional protocols require sequential acquisitions for multi‐breath‐hold 2D cine and 3D MR angiography (MRA), which is time‐consuming. In addition, breath‐hold 2D cine can be challenging for patients with limited breath‐hold capacity.

**Purpose:**

To further develop and evaluate a free‐breathing 3D whole‐heart cine MRI technique for simultaneous assessment of cardiac function and aortic anatomy in a single non‐contrast acquisition at 1.5 T.

**Study Type:**

Prospective.

**Subjects:**

Twenty‐four healthy volunteers (mean age 31.8 ± 9.9 years, 50% female).

**Field Strength/Sequences:**

1.5 T; A cartesian spiral (CASPR) bSSFP 3D cine, 2D cine and 3D mDixon MRA.

**Assessment:**

Acquisition and reconstruction times were assessed for 3D cine at 2.5 and 2.0 mm. LV mass, LVEF, and RVEF were assessed by two observers (5 and 32 years of experience) and compared with 2D cine. Aortic root and ascending aortic areas were compared with 3D MRA. Image quality was evaluated using blood pool‐to‐myocardium contrast ratio and endocardial/epicardial edge sharpness. Qualitative image preference was assessed by three observers (5, 32, and 33 years of experience).

**Statistical Tests:**

One‐way ANOVA with Tukey post hoc tests and Bland–Altman analysis with paired *t*‐tests were used. Intraclass correlation coefficient (ICC) assessed inter‐ and intra‐observer agreement. *p* < 0.05 was considered significant.

**Results:**

Acquisition time was 5 min (2.5 mm) and 7 min (2 mm), versus 11 min for 2D cine and 8 min for 3D MRA. Reconstruction time was approximately 5 min. LV mass showed no differences versus 2D cine. LVEF showed small but significant bias (2.5 mm: 1.38%, *p* = 0.005; 2.0 mm: 1.39%, *p* < 0.001). RVEF showed no significant differences. Ascending aorta areas showed significant differences (0.28mm^2^ and 0.25mm^2^, *p* < 0.001), while aortic root areas showed no difference versus 3D MRA. Reproducibility was at least moderate (ICC 0.75–0.998). 3D cine showed lower contrast and edge sharpness than 2D cine (p < 0.001). Observers preferred 2.0 mm (ICC = 0.64).

**Data Conclusion:**

Free‐breathing 3D cine MRI enables operator‐independent, time‐efficient, and accurate assessment of cardiac function and aortic anatomy in healthy volunteers in a single non‐contrast acquisition at 1.5 T, compared to conventional 2D cine and 3D MRA.

**Evidence Level:**

2.

**Technical Efficacy:**

Stage 1.

## Plain Language Summary

Cardiac MRI typically requires multiple separate scans performed while patients hold their breath, which is time‐consuming and challenging for some patients. This study evaluated a new free‐breathing 3D MRI technique that simultaneously assesses heart function and aortic anatomy in a single 5–7 min scan without a contrast agent injection. Tested in 24 volunteers, the technique produced measurements of heart function and aortic size that were comparable to conventional methods, while being faster and easier for patients. This approach could simplify cardiac MRI examinations and make them more accessible in routine clinical practice.

## 1 Introduction

Cardiovascular diseases are the leading cause of morbidity and mortality worldwide, imposing a substantial burden on healthcare systems [1]. Cardiovascular MRI has excellent soft‐tissue contrast and is the reference standard for noninvasive assessment of cardiovascular morphology and function [2]. However, conventional multi‐slice breath‐hold 2D cine MRI may be difficult for patients and requires technologist training for precise geometric planning. Prolonged planning times, multiple breath‐holds, and slice misalignments due to variable breath‐hold levels complicate its routine use. Although 2D real‐time cine MRI allows continuous image acquisition during free breathing [3, 4, 5, 6], it provides only anisotropic resolution, requires multiple views, and is not readily quantifiable with standard post‐processing tools.

Assessment of aortic morphology and dilation is important in aortic diseases such as aneurysm and dissection [7]. Free‐breathing 3D modified Dixon (mDixon) MR angiography (MRA), with respiratory motion correction via pencil‐beam navigation, is a contrast‐free technique which is regarded as a clinical reference standard for aortic quantification [8, 9]. However, it captures only a single cardiac phase, usually the end‐diastole.

Recently, free‐breathing (or free‐running) 3D cine cardiac MRI has enabled the acquisition of volumetric cine data in a single acquisition lasting several minutes [10, 11]. Images can be reconstructed and reformatted into any desired view (dynamic multiplanar reformatting), removing the need for complex planning and multiple breath‐holds. This approach may benefit patients who struggle with breath‐holding, ensures slice continuity in space and time, and supports automation to address the growing demand for cardiac MRI.

The contrast mechanism of cine cardiac MRI is typically that of a balanced steady state free precession sequence (bSSFP) acquisition due to its superior signal‐to‐noise ratio and blood‐myocardium contrast‐to‐ratio [12]. Previous respiratory motion compensation techniques, such as navigator gating, have demonstrated effective motion suppression. However, they reduce imaging efficiency by discarding data outside a predefined gating window [13]. In contrast, self‐gating derives respiratory motion from k‐space, allowing efficient, continuous motion‐corrected acquisition without external surrogates [14]. In terms of k‐space filling strategies that are favorable both to under sampling and self‐navigation, previous studies have reported acquisition methods based on:

1. Radial (non‐cartesian) sampling in 3D k‐space, such as radial “spiral” phyllotaxis [15], radial stack of‐stars [16, 17] and koosh‐ball [11, 18].
Cartesian sampling in 3D k‐space, such as MUSIC [19, 20], centric reordering [21], and CASPR [10, 22]. CASPR (Cartesian Acquisition with Spiral Profile Reordering) was initially proposed by Doneva et al. [23] and further developed by Prieto et al. [24].

Radial non‐cartesian reconstructions with large amounts of data are computationally expensive due to the need for re‐gridding. A modified CASPR trajectory employing a spiral‐in/spiral‐out design with k_0_ acquired at the centre of each shot to eliminate large k‐space discontinuities before and after readout, combined with a fully deformable motion model for respiratory compensation in the reconstruction has been proposed as a more efficient alternative [25]. However, its feasibility and diagnostic performance have not yet been evaluated in a clinical setting, representing a critical gap between methodological development and clinical translation.

Thus the aim of this study was to further develop a non‐contrast CASPR free‐breathing isotropic cardiovascular 3D cine bSSFP sequence based on preliminary work, deploy it on the MR scanner, and evaluate it against clinical standard multi‐slice, multi‐breath‐hold 2D cine acquisition, and 3D mDixon MRA, using quantitative parameters related to cardiac function, cardiac anatomy, and aortic anatomy.

## 2 Methods

### 2.1 Participants

This study was approved by the institutional review board and performed in accordance with the Declaration of Helsinki. Twenty four volunteers were prospectively recruited from September 2024 to January 2025 and gave written informed consent before inclusion. Volunteers older than 18 years without MRI contraindications (e.g., claustrophobia, pacemakers) were eligible for inclusion.

### 2.2 MR Imaging Acquisition

All volunteers were scanned on a 1.5 T MRI scanner (Ingenia, Philips Healthcare, Best, The Netherlands) using a 28‐channel anterior/posterior array coil. Conventional multi‐slice 2D cine short‐axis images were acquired using ECG‐triggered bSSFP in end‐expiration, along with single‐slice two‐ and four‐chamber views (flip angle 60°, slice thickness 8 mm, TE/TR 1.27/2.5 ms, field of view (FOV) 350 × 350 × 112 mm^3^, voxel size 1.5 × 1.51 × 8.0 mm^3^).

The 3D mDixon MRA was obtained using a pencil‐beam navigator‐gated, non‐contrast, ECG‐gated protocol (TR/TE 3.2/1.07–1.9 ms, flip angle 20°, FOV 300 × 300 × 125 mm^3^, voxel size 2.0 × 1.98 × 2.0 mm^3^), acquiring dual‐echo images for fat–water separation.

**FIGURE 1:**
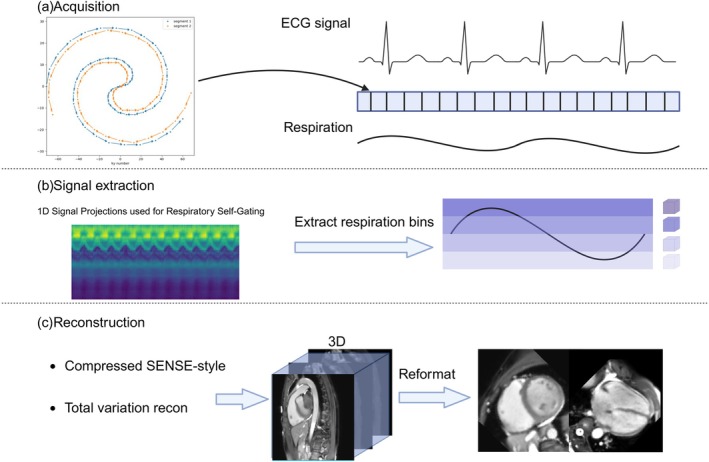
Flowchart of acquisition and reconstruction methods. (a) CASPR segments of k‐space are acquired continuously, and each line of k‐space is labeled with the cardiac phase time using the ECG signal. (b) Each segment acquires the center line of k‐space which enables respiratory self‐navigation via binning. (c) The ECG and respiratory binned data is reconstructed to a 3D cine dataset, using a total variation compressed SENSE‐style recon and then manually reformatted to clinical reference multi‐slice 2D cine views.

The free‐breathing 3D cine sequence was planned in a few seconds by placing a 3D sagittal volume without angulations on multi‐stack survey views. Slice coverage was optimized to include the entire heart and aortic arch with sufficient margin for respiratory displacement. The sequence was acquired twice per volunteer at 2.5 mm and 2.0 mm isotropic resolution. A bSSFP sequence includes abbreviated binomial [1] water‐selective pulses [26], which keeps the TR short and hence keeps the bSSFP off‐resonance dark band artifacts far from the region of interest. The imaging parameters included: The imaging parameters included: FOV = 280 × 280 mm^2^ (AP × FH), with the RL dimension adjusted per subject to ensure full cardiac coverage, TR/TE = 3.8/1.9 ms and flip angle = 50°, voxel size was 2.5 × 2.5 × 2.5 mm^3^ for 2.5 mm isotropic resolution and 2.0 × 2.0 × 2.0 mm^3^ for 2.0 mm isotropic resolution. The CASPR‐based method samples 3D k‐space continuously in time (k‐t) with segments consisting of concatenations of inward‐outward spirals in the k_y_ and k_z_ plane (Figure 1a, the dots of each segment represent the crossing of each k_x_ line of k‐space with the k_y_‐k_z_ plane), that rotate with tiny golden‐angle increments from segment to segment (2 examples of such segments are shown in 2 different colors in Figure 1a). Every segment acquires the central line of 3D k‐space every 190 ms, which is sufficient for respiratory self‐gating. The smooth k‐space trajectory minimizes eddy‐current effects caused by gradient switching [27]. A standard ECG signal recorded cardiac phase timing.

### 2.3 MR Image Reconstruction

The reconstruction was performed offline on a Windows Server 2022 system equipped with NVIDIA A10 GPU using NVIDIA CUDA drivers for GPU acceleration. Reconstruction consists of 4 essential steps. Firstly, respiratory self‐navigation was performed using the 1D projection in the feet‐head direction obtained from the k = 0 profiles measured in each shot/segment (Figure 1). Singular‐value decomposition (SVD) in combination with a band‐pass filter to select respiratory frequencies [28] was used to compute a respiratory signal and used together with the ECG signal to sort the measured data into 4 respiratory and multiple cardiac motion bins [10, 29].

Secondly, the respiratory state corresponding to the end‐expiratory position (reference state) was reconstructed using total‐variation (TV) in the cardiac phase dimension by minimizing:
(1)

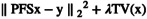

Here, the final image, x, contains the 3D volume and the cardiac phase dimension, P the projection operator, S represents the coil‐sensitivity maps obtained from a sensitivity‐encoding reference scan, F the Fourier Transform. Averaging all cardiac phases, images for each respiratory motion state were reconstructed using five Conjugate‐Gradient iterations.

As third step, deformation vector fields (DVF) between reference state and other respiratory motion states were computed from the respiratory‐resolved images by minimizing the mean‐square‐error using gradient‐descent. DVF and inverse DVF were computed simultaneously using a diffeomorphic deformation model based on static flow fields [30] using backpropagation and additionally regularized by a cycle loss and a quadratic gradient penalty [25]. Deformable registration between the respiratory motion states allowed the use of nearly all measured data.

Finally, the under sampled multi‐channel data from each cardiac phase were reconstructed using a motion‐compensated iterative reconstruction. As k‐space was continuously filled by concatenations of inward‐outward spirals that rotate with tiny golden‐angle increments in the presence of continuous respiratory motion and cardiac motion, this resulted in an incoherent subsampling pattern across cardiac and respiratory motion states, which lends itself to robust compressed sensing‐style total variation rapid GPU‐based iterative reconstruction. For this, the cost function:
(2)

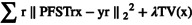

is minimized using the algorithm that is specialized to temporal TV [31]. Here, the operator Tr (from DVF Step 2) morphs × into the image series corresponding to respiratory state r, and yr is the associated multi‐coil, binned k‐space data, which is measured at locations indicated by the projection operator P [25].

### 2.4 Validation Experiments

3D cine data were manually reformatted into multi‐slice 2D short‐axis views following a predefined protocol consistent with standard 2D short‐axis planning, with slice thickness matched to that of conventional 2D cine imaging (8 mm). Left ventricular (LV) mass, LV ejection fraction (EF), and right ventricular (RV) EF were measured using Medis Suite (Medis, Leiden, The Netherlands). Contours were automatically generated via a deep learning algorithm with manual corrections when needed. Aortic root (AR) and ascending aorta (AAo) cross‐section areas were measured using navigated 3D MRA and matching views from the free‐breathing 3D cine images via Medis Suite 3D View application. Measurements were performed by two readers (RC and JW, with 5 and 32 years of experience, respectively) and interobserver agreement assessed. Intra‐observer agreement was evaluated by repeat analysis from RC, with a three month interval between assessments.

### 2.5 Image Quality Evaluation

Quantitative assessment included LV blood pool‐to‐myocardium contrast ratio (CR) and endocardial/epicardial edge sharpness (10%–90% rise distance, RD) using MASS (Leiden University Medical Center, Leiden, The Netherlands).

CR assessment involved manually delineating a contour in the mid‐interventricular septum as the first region of interest (ROI), copying this contour, and subsequently moving it to the LV blood pool as the second ROI while avoiding the papillary muscles and trabeculations. Mean signal intensities (SI) from both ROIs were measured to calculate blood pool‐to‐myocardium contrast ratio:

(3) $$\mathrm{CR}=\frac{\text{mean}\left(\mathrm{SI}\;\text{blood}\right)-\text{mean}\;\left(\mathrm{SI}\;\text{myocardium}\right)}{\text{mean}\;\left(\mathrm{SI}\;\text{myocardium}\right)}$$

Edge sharpness of the endocardium and epicardium was measured by drawing a straight mid‐septal line perpendicular to the interventricular septum. This line extended from the RV blood pool to the LV blood pool on the same mid‐ventricular short‐axis view slice at end‐diastole for each participant. A line signal intensity graph was then exported, and the 10%–90% RD of the endocardium and epicardium edges was calculated using an in‐house developed tool. A shorter RD indicates a more rapid increase in intensity, which reflects higher edge sharpness.

A blinded qualitative comparison was performed by presenting 3D cine images at 2.5 mm and 2.0 mm resolutions side‐by‐side in randomized, anonymized order. Three readers (RC, HL, and JW, with 5, 33, and 32 years of experience, respectively) were asked to select their preferred dataset based on contrast, artifacts, and endocardial edge clarity.

### 2.6 Statistical Analysis

One‐way ANOVA with Tukey post hoc tests evaluated differences between free‐breathing 3D cine (2.5 mm and 2.0 mm) and multi‐breath‐hold 2D cine datasets. Bland Altman analysis was also performed with mean difference (bias) and 95% limits of agreement (LoA), and a paired *t*‐test was used to evaluate statistical significance of the differences.

Intra‐observer and inter‐observer agreement for ventricular and aortic measurements was assessed using the intraclass correlation coefficient (ICC), with ICC < 0.50 regarded as poor, ICC 0.50–0.75 regarded as moderate, ICC > 0.75–0.90 regarded as good, and ICC > 0.9 regarded as excellent [32]. Inter‐observer agreement for the qualitative, preference‐based comparison of 3D cine images was assessed using the ICC.

Statistical analyses were performed using SPSS (v29.0) with significance set at *p* < 0.05; figures were generated in GraphPad Prism (v10.2.3).

## 3 Results

Multi‐slice, multi‐breath‐hold 2D cine images were acquired in 24 volunteers (12 (50%) female; mean age, 31.8 ± 9.9 years old), all of whom had no history of arrhythmia or cardiac surgery. 3D mDixon MRA images were obtained in a subset of 21 volunteers; the remaining three volunteers did not undergo 3D mDixon MRA due to scan time constraints. The baseline characteristics of the volunteers are shown in Table 1. Total planning and acquisition time for conventional 2D cine, including 3 long‐axis and 1 short‐axis views, averaged 11 min (range 9–15 min), while 3D mDixon MRA averaged 8 min (range 6–13 min).

**TABLE 1:** Baseline volunteer characteristics.

Characteristics	All participants (*n* = 24)
Age (year)	31.8 ± 9.9
Sex, female (%)	12 (50%)
Height (cm)	172.4 ± 10.5
Weight (kg)	68.2 ± 16.8
Body mass index (kg/m^2^)	22.6 ± 3.2

*Note:* Data are means ± SDs.

The k‐space data for free‐breathing 3D cine were acquired and reconstructed successfully for all 24 volunteers (24 × 2 acquisitions = 48 data sets). The planning acquisition required an average of 30 s. However, due to the varying number of slices required to adapt to the size of the subject's heart, the total acquisition time varied across volunteers, with an average acquisition time of approximately 5 min (range 4–7 min) for the 2.5 mm isotropic resolution, and 7 min (range 5–9 min) for the 2 mm isotropic resolution, respectively. The GPU‐based reconstruction time was approximately 5 min per dataset.

**FIGURE 2:**
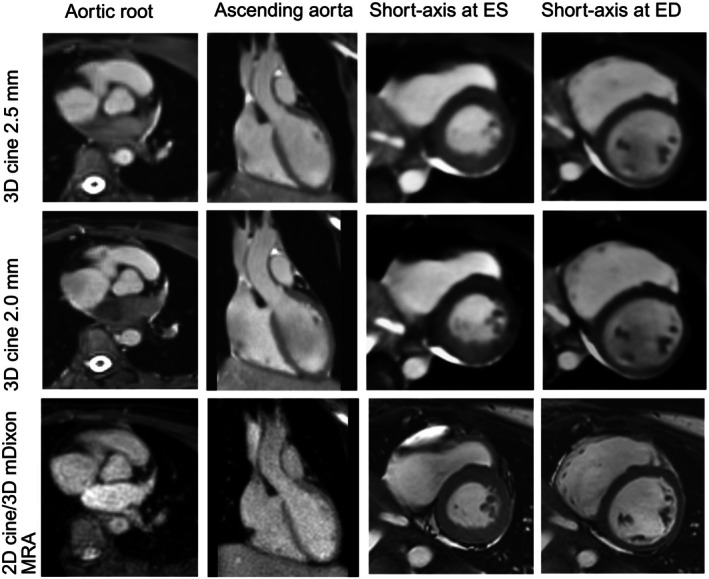
Comparison of free‐breathing 3D cine at 2.5 mm and 2.0 mm isotropic resolution, multi‐breath‐hold 2D cine, and 3D mDixon MRA in a 27‐year‐old female volunteer. The aortic root is shown in axial view and the ascending aorta in coronal view. Short‐axis views are displayed at both end‐systolic (ES) and end‐diastolic (ED) phases.

Figure 2 shows typical image quality of free‐breathing 3D cine 2.5 mm and 2.0 mm isotropic acquisitions, as well as the reference clinical standard multi‐breath‐hold 2D cine images.

**FIGURE 3:**
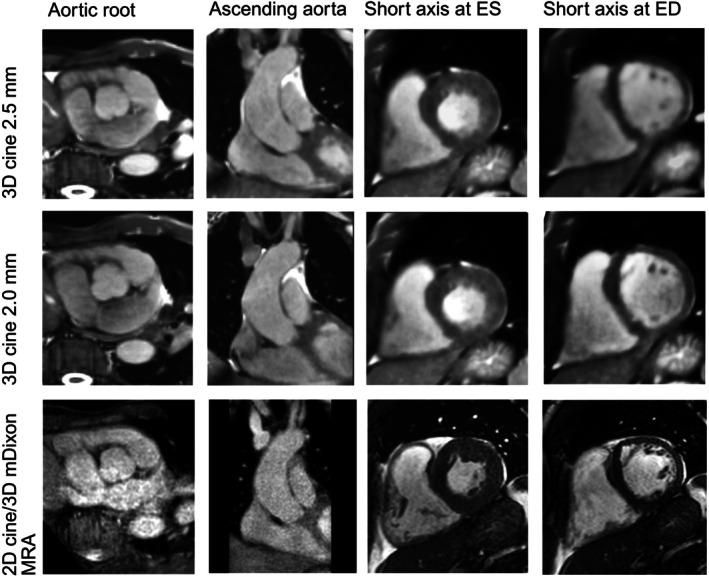
Comparison of free‐breathing 3D cine at 2.5 mm and 2.0 mm isotropic resolution, multi‐breath‐hold 2D cine, and 3D mDixon MRA in a 64‐year‐old male volunteer with dilated ascending aorta. The aortic root is shown in axial view and the ascending aorta in coronal view. Short‐axis views are displayed at both end‐systolic (ES) and end‐diastolic (ED) phases.

One of the volunteers was found to have a dilated aorta (Figure 3).

### 3.1 Cardiac and Aortic Measurements

**FIGURE 4:**
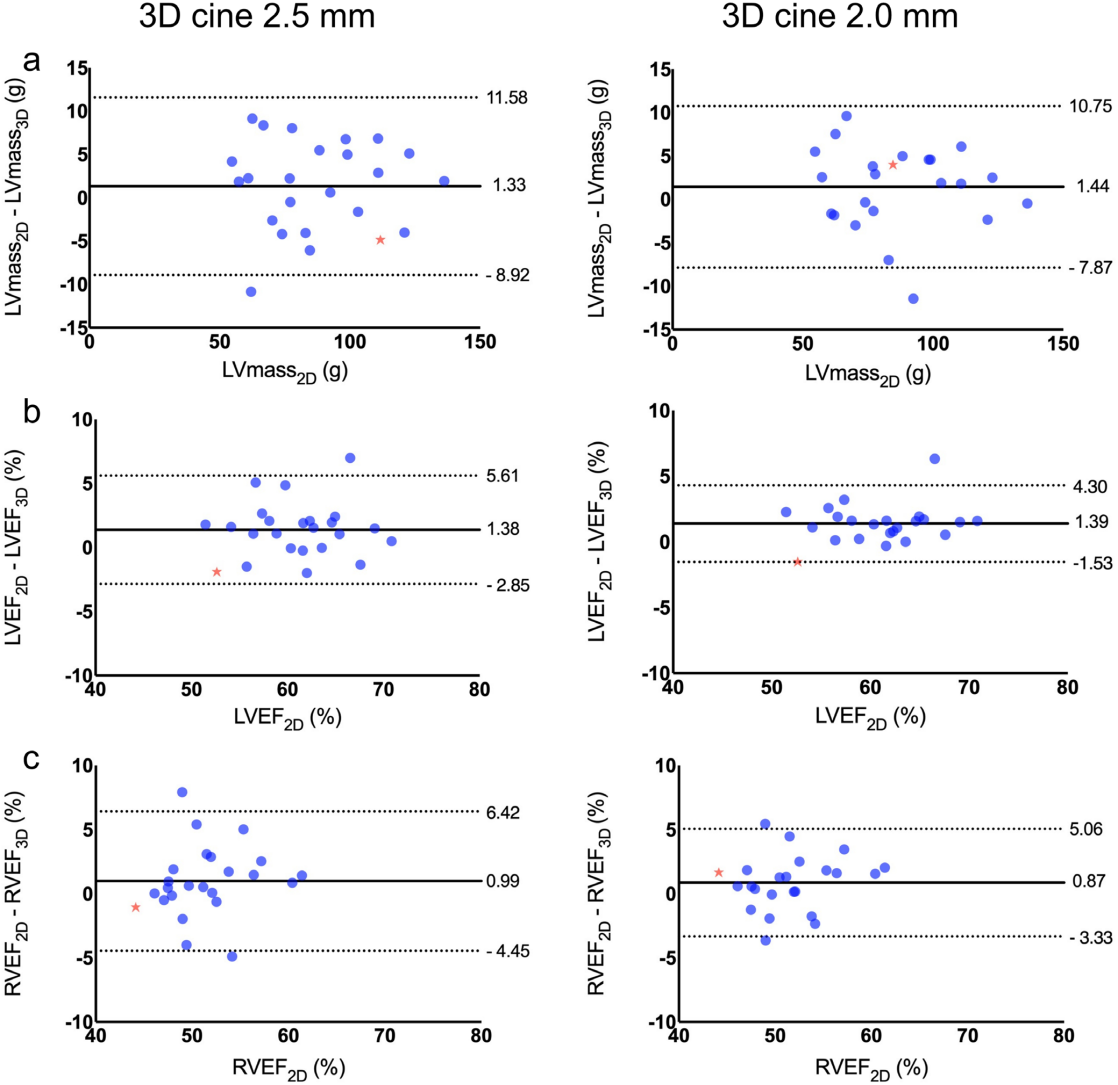
Bland–Altman analysis comparing free‐breathing 3D cine at 2.5 mm and 2.0 mm isotropic resolution with multi‐breath‐hold 2D cine. Solid lines indicate mean differences, and dashed lines indicate the 95% limits of agreement (LoA). (a) Left ventricle (LV) mass measured on short‐axis views. (b) LV ejection fraction (EF) measured on short‐axis views. (c) Right ventricle EF measured on short‐axis views. The red star represents a volunteer with a dilated aorta, blue dots represent other volunteers.

The LV mass measurements between free‐breathing 3D cine 2.5 mm and multi‐breath‐hold 2D cine showed a mean difference of 1.33 g, with the 95% LoA ranging from −8.92 g to 11.58 g. This difference was not statistically significant (*p* = 0.23). Similar agreement was observed in LV mass measurements between free‐breathing 3D cine 2.0 mm and multi‐breath‐hold 2D cine, with a mean difference of 1.44 g, which was also not statistically significant (*p* = 0.16). The 95% LoA for the free‐breathing 3D cine 2.0 mm (−7.87 g to 10.75 g) was narrower than those of free‐breathing 3D cine 2.5 mm (Figure 4a).

Bland–Altman analyses showed a mean difference of 1.38% (*p* = 0.005) in LVEF measurements between free‐breathing 3D cine 2.5 mm and multi‐breath‐hold 2D cine, and a mean difference of 1.39% (*p* < 0.001) between free‐breathing 3D cine 2.0 mm and multi‐breath‐hold 2D cine. The 95% LoA were narrower for free‐breathing 3D cine 2.0 mm (−1.53% to 4.30%) than for free‐breathing 3D cine 2.5 mm (−2.85% to 5.61%) (Figure 4b).

There was no significant difference between the free‐breathing 3D cine 2.5 mm RVEF and the multi‐breath‐hold 2D cine RVEF, with a mean difference of 0.99% (*p* = 0.09) and 95% LoA ranging from −4.45% to 6.42%. Similarly, the RVEF measurements between free‐breathing 3D cine 2.0 mm and multi‐breath‐hold 2D cine were not significantly different, with a mean difference of 0.87% (*p* = 0.07) and 95% LoA ranging from −3.33% to 5.06%. The 95% LoA for free‐breathing 3D cine 2.0 mm were narrower than those for free‐breathing 3D cine 2.5 mm (Figure 4c).

**FIGURE 5:**
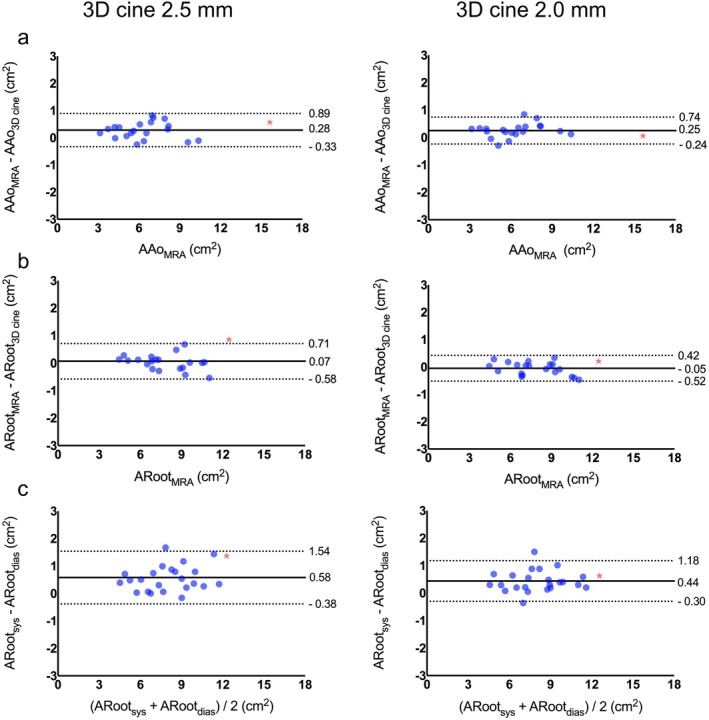
Bland–Altman analysis comparing free‐breathing 3D cine at 2.5 mm and 2.0 mm isotropic resolution with 3D mDixon MRA. Solid lines indicate mean differences, and dashed lines indicate the 95% limits of agreement (LoA). (a) Ascending aorta (AA) measured at the end‐diastole phase. (b) Aortic root (AR) measured at the end‐diastolic phase. (c) Aortic root (AR) measured at both the end‐systolic and end‐diastolic phase within free‐breathing 3D cine at both 2.5 mm and 2.0 mm isotropic resolution. The red star represents a volunteer with a dilated aorta, blue dots represent other volunteers.

Ascending aorta cross‐sectional areas measured with the free‐breathing 3D cine 2.5 mm and 3D mDixon MRA techniques were not significantly different, with a mean difference of 0.28 mm^2^ (*p* < 0.001), and 95% LoA ranging from −0.33 mm^2^ to 0.89 mm^2^ (Figure 5a). Similarly, the mean difference between free‐breathing 3D cine 2.0 mm and 3D mDixon MRA ascending aorta cross‐sectional area was 0.25 mm^2^ (*p* < 0.001), with 95% LoA ranging from −0.24 mm^2^ to 0.74 mm^2^ (Figure 5a).

Aortic root cross‐sectional areas measured with free‐breathing 3D cine 2.5 mm and 3D mDixon MRA were not significantly different, with a mean difference of 0.07 mm^2^ (*p* = 0.38), while the 95% LoA ranged from −0.58 mm^2^ to 0.71 mm^2^ (Figure 5b). The aortic root cross‐sectional areas were similarly in good agreement between free‐breathing 3D cine 2.0 mm and 3D mDixon MRA with a mean difference of −0.05 mm^2^ (*p* = 0.33), and 95% LoA ranged from −0.52 mm^2^ to 0.42 mm^2^ (Figure 5b).

On the free‐breathing 3D cine images, aortic root cross‐sectional areas at end‐systole were significantly larger than those at end‐diastole, with a mean difference of 0.58 mm^2^ (*p* < 0.001) in free‐breathing 3D cine 2.5 mm images and 0.44 mm^2^ (*p* < 0.001) in free‐breathing 3D cine 2.0 mm images. (Figure 5c).

All measurements demonstrated at least moderate intra‐ and inter‐observer agreement (Table 2). To improve clarity and avoid misinterpretation due to rounding, ICC values greater than 0.95 are reported to three decimal places.

**TABLE 2:** Intra‐ and inter‐observer agreement in left ventricular (LV) mass, LV ejection fraction (EF), right ventricular (RV) EF, and ascending and root aorta cross‐sectional areas.

	Sequence	Inter‐observer agreement		Intra‐observer agreement	
		ICC	95% CI	ICC	95% CI
LV mass	2D cine	0.998	0.995–0.999	0.994	0.986–0.997
	3D cine 2.5 mm	0.989	0.974–0.995	0.981	0.955–0.992
	3D cine 2.0 mm	0.991	0.979–0.996	0.965	0.917–0.985
LVEF	2D cine	0.986	0.968–0.994	0.974	0.940–0.989
	3D cine 2.5 mm	0.953	0.891–0.980	0.985	0.965–0.993
	3D cine 2.0 mm	0.88	0.71–0.95	0.970	0.928–0.987
RVEF	2D cine	0.86	0.68–0.94	0.95	0.88–0.98
	3D cine 2.5 mm	0.89	0.75–0.95	0.94	0.85–0.97
	3D cine 2.0 mm	0.75	0.41–0.89	0.86	0.66–0.94
AR	3D MRA	0.988	0.972–0.995	0.995	0.989–0.998
	3D cine 2.5 mm_dias	0.985	0.966–0.994	0.990	0.977–0.996
	3D cine 2.0 mm_dias	0.986	0.967–0.994	0.990	0.977–0.996
	3D cine 2.5 mm_sys	0.994	0.986–0.998	0.995	0.989–0.998
	3D cine 2.0 mm_sys	0.990	0.976–0.995	0.994	0.985–0.997
AA	3D MRA	0.990	0.977–0.996	0.996	0.991–0.998
	3D cine 2.5 mm_dias	0.989	0.975–0.995	0.993	0.983–0.997
	3D cine 2.0 mm_dias	0.989	0.975–0.995	0.995	0.987–0.998

Abbreviations: Sys, systole; dias, diastole.

### 3.2 Image Quality Evaluation

**FIGURE 6:**
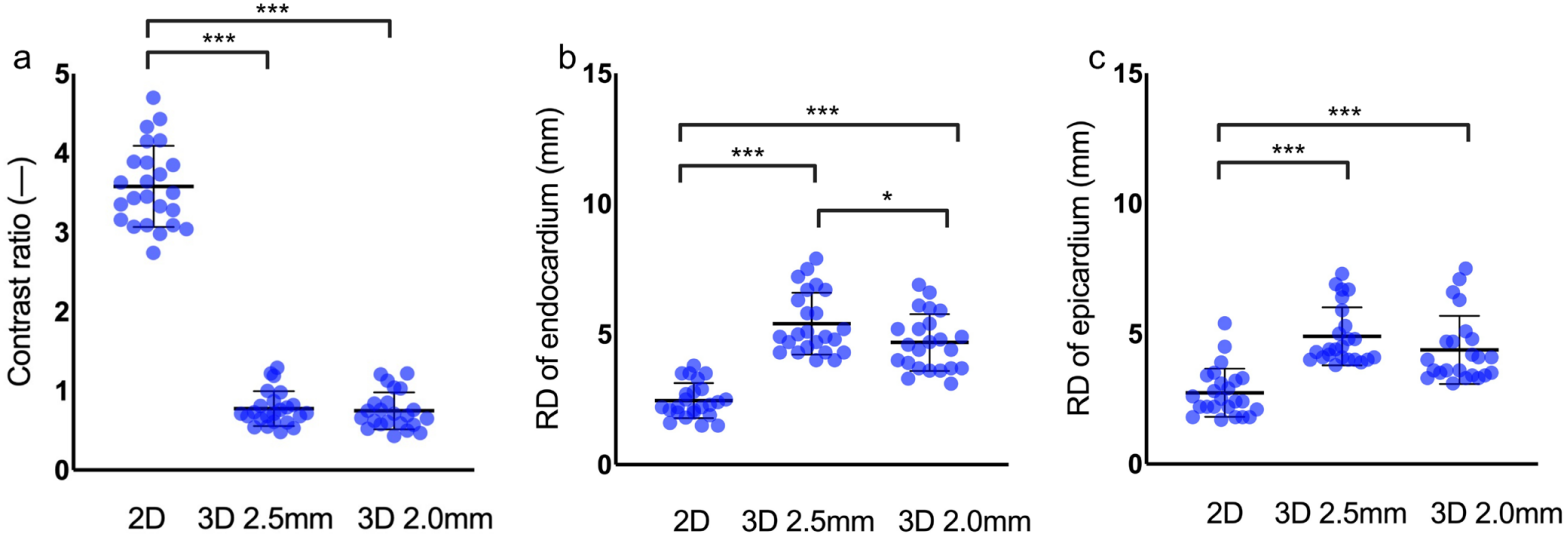
Comparison of imaging quality parameters among free‐breathing 3D cine at 2.5 mm and 2.0 mm isotropic resolution and multi‐breath‐hold 2D cine. (a) Contrast ratio of blood pool to myocardium. (b) Rise distance of endocardium. (c) Rise distance of epicardium. Statistical significance computed with one‐way ANOVA with Tukey post hoc tests is indicated with the brackets, * for *p* < 0.05, *** for *p* < 0.001.

The free‐breathing 3D cine images demonstrated sufficient blood‐pool‐to‐myocardium contrast for LV segmentation, with a CR of 0.8 ± 0.2 at 2.5 mm isotropic resolution and 0.7 ± 0.3 at 2.0 mm isotropic resolution. One‐way ANOVA with Tukey post hoc tests showed that the CRs of free‐breathing 3D cine images were significantly lower than that of multi‐breath‐hold 2D cine images, which had a CR of 3.6 ± 0.5 (Figure 6a,p < 0.001). There was no significant difference in CR between free‐breathing 3D cine 2.5 mm and 2.0 mm isotropic resolution (Table 3, *p* = 0.86).

**TABLE 3:** Imaging quality measurements for free‐breathing 3D cine at 2.5 mm and 2.0 mm isotropic resolution, and multi‐breath‐hold 2D cine.

	3D cine 2.5 mm	3D cine 2.0 mm	2D cine	*p*
Mean Contrast ratio (%)	0.8 ± 0.2	0.7 ± 0.3	3.6 ± 0.5^a^, ^b^	< 0.001
Mean RD of endocardium (mm)	5.4 ± 1.2^b^	4.7 ± 1.1	2.5 ± 0.7^a^, ^b^	< 0.001
Mean RD of epicardium (mm)	4.9 ± 1.1	4.4 ± 1.3	2.7 ± 0.9^a^, ^b^	< 0.001

*Note:* Data are expressed as means ± standard deviations. The *p* value shows the ANOVA test between the three groups.

Abbreviation: RD, rise distance.

^a^ Post hoc significant difference versus free‐breathing 3D cine 2.5 mm (*p* < 0.05).

^b^ Post hoc significant difference versus free‐breathing 3D cine 2.0 mm (*p* < 0.05).

The sharpness of LV endocardium was significantly lower for free‐breathing 3D cine images at 2.5 mm isotropic resolution, with a RD of 5.4 ± 1.2 mm, as compared to free‐breathing 3D cine images at 2.0 mm isotropic resolution, which had an RD of 4.7 ± 1.1 mm (*p* = 0.041). Multi‐breath‐hold 2D cine images had significantly higher sharpness, with the lowest RD of 2.5 ± 0.7 mm (Figure 6b, Table 3, *p* < 0.001). The sharpness of the LV epicardium in free‐breathing 3D cine images was significantly lower than that in multi‐breath‐hold 2D cine images, with RD of 4.9 ± 1.1 mm at 2.5 mm isotropic resolution and 4.4 ± 1.3 mm at 2.0 mm isotropic resolution, as compared to 2.7 ± 0.9 mm for the multi‐breath‐hold 2D cine images (Figure 6c,p < 0.001). However, there was no significant difference in RD between free‐breathing 3D cine images at 2.5 mm and 2.0 mm (Table 3, *p* = 0.26).

Blinded qualitative assessment showed that free‐breathing 3D cine images at 2.0 mm isotropic resolution were preferred for overall imaging quality as compared to those acquired at 2.5 mm isotropic resolution. Observer 1 selected 2.0 mm in 88% of cases, observer 2 in 92% of cases, and observer 3 in 80% of cases. Inter‐observer agreement for the qualitative, preference‐based comparison of 3D cine images among three observers was moderate, with an ICC of 0.64.

## 4 Discussion

A free‐breathing isotropic cardiovascular 3D cine MRI approach was developed and tested at 1.5 T in volunteers without use of contrast agent. Free‐breathing 3D cine MRI provides quantitative cardiovascular function and aortic anatomy consistent with conventional multi‐breath‐hold 2D cine and 3D mDixon MRA. Image quality was sufficient for cardiovascular assessments across all subjects.

The endocardium/epicardium sharpness and blood/myocardium contrast were significantly better for multi‐breath‐hold 2D cine acquisitions than for the 3D acquisitions. This is likely due to the acquired in plane pixel size being superior for the 2D acquisitions and there being a reduced inflow effect in free‐breathing 3D cine as compared to multi‐breath‐hold 2D cine imaging [33]. The visual and quantitative evaluation of the proposed free‐breathing 3D cine approach nevertheless showed sufficient quality for quantitative assessment, in combination with a largely improved workflow and patient comfort level.

Cardiovascular MRI is performed widely using 1.5 T MRI systems, which offer a balanced combination of image quality, patient safety, and widespread clinical availability [34]. The free‐breathing 3D cine MRI technique developed at 1.5 T eliminates the need for breath‐holds and thereby improves patient comfort while providing cardiac measurements which did not differ significantly from those obtained with multi‐breath‐hold 2D cine imaging. Together with previous free‐breathing 3D cine studies conducted at various field strengths [35, 36, 37], the present work further supports the broad feasibility and clinical applicability of contrast‐free free‐breathing cardiac imaging across a range of field strengths.

In this healthy volunteer study, free‐breathing 3D cine acquisition times of 5–7 min (depending on resolution) provided good image quality and accurately captured both respiratory and cardiac motion. Increasing the total amount of 3D k‐t space lines acquired by the 3D cine would not only improve the signal‐to‐noise ratio, but also make the acquisition more robust to ECG variations/arrhythmias and respiratory variations. However, that would come at the cost of increasing the total scan time of the sequence, which in turn would lead to potential patient discomfort. Our initial experience in healthy volunteers suggests that 5–7 min acquisitions are appropriate and were well tolerated.

Respiratory motion compensation in free‐breathing whole heart cine reconstruction has been limited to rigid or affine transformation models, which are insufficient to fully describe the complex, non‐rigid deformations of the heart during breathing [22, 23, 24]. A key strength of this study is the incorporation of a fully deformable motion model, enabling more accurate and comprehensive compensation of non‐rigid respiratory motion. The current reconstruction time of around 5 min represents a substantial improvement over previously proposed frameworks that require longer reconstruction times [10, 35, 38], thereby improving clinical feasibility. Although the reconstruction was performed offline due to hardware constraints at the time of acquisition, the pipeline is fully equivalent to the inline implementation now available on current scanner hardware, with a new version achieving reconstruction times of the order of 90 s scheduled for use in future patient studies, meeting the requirements of routine clinical care.

This initial volunteer study demonstrated key advantages of the proposed method. Firstly, the planning duration was approximately 30 s and can be performed by technicians without any special cardiac MRI training. Secondly, free‐breathing acquisition improved subject comfort and minimized respiratory motion artifacts typically associated with conventional multi‐breath‐hold 2D cine imaging [39]. Thirdly, free‐breathing 3D cine data is richer in clinical information than multi‐breath‐hold 2D data. Lastly, the overall acquisition protocol duration for the new free‐breathing 3D method is shorter and less variable than that of the multi‐breath‐hold 2D approach, which requires a time‐consuming multi‐view, multi‐breath hold geometrical planning stage.

In this study, there was a clinically acceptable underestimation of biventricular measurements in free‐breathing 3D cine images, which is consistent with previous studies [35, 36]. The exact causes of systematic bias remain unknown; however, several potential reasons may contribute. Firstly, because free‐breathing 3D cine images were reformatted into conventional 2D short‐axis views for cardiac function measurements, slight mismatches in anatomical slice locations may occur. Secondly, the lower blood‐myocardium contrast and reduced in‐plane resolution of free‐breathing 3D cine images compared with multi‐breath‐hold 2D cine [22, 33, 35, 40] likely contributed to the observed variability in biventricular contour delineation.

Future studies comparing free‐breathing 3D cine and multi‐breath‐hold 2D cine should also take into account the potential misalignment between subsequent breath‐hold positions that can occur with multiple breath‐holding, as previously reported [41]. Although shearing of multi‐breath‐hold 2D short‐axis slices (relative parallel displacements) does not affect the ejection fraction measurement, the associated through‐plane motion effect cannot be corrected in post processing and becomes “invisible”. This limitation of multi‐breath‐hold 2D cine can negatively impact volumetric calculations [42] and diagnostic assessment [43]. While free‐breathing 3D cine avoids irreproducible breath‐holding, future studies are needed to confirm this advantage.

Future work should also contribute to exploring more efficient segmentation methods in 3D to fully leverage the benefits of free breathing 3D cine technique, such as a 3D approach for quantitative measurement with appropriate 3D post‐processing methods.

In this study, free‐breathing 3D cine imaging enabled assessment of aortic dynamics throughout the cardiac cycle and showed good agreement with reference 3D mDixon MRA. Although a statistically significant difference was observed for ascending aorta cross‐sectional area, the absolute bias remained small and clinically acceptable. As expected, aortic root area was larger at end‐systole compared to end‐diastole. Segmenting the aorta in 3D reduces angulation‐related variability and allows for a more comprehensive assessment. Ooij et al. [44], for example, have applied a free‐running 3D cine sequence in combination with AI segmentation to quantify aortic displacement and diameters in healthy volunteers. However, clinical studies involving patients are limited, and further exploration of aortic post‐processing methods based on free‐breathing 3D cine is needed.

### 4.1 Limitations

This study is limited by its small sample size, which restricts statistical power, and by exclusive focus on volunteers with normal sinus rhythm and regular breathing patterns. To establish clinical reliability, future validation should involve larger cohorts that include patients with arrhythmias, erratic respiration, and a spectrum of cardiac diseases. Furthermore, only a limited set of cardiac functional metrics was evaluated. Additional clinically relevant parameters should be included in future studies to more comprehensively validate the proposed method.

## 5 Conclusion

In healthy volunteers, free‐breathing isotropic cardiovascular 3D cine MRI with cartesian spiral sampling, performed without contrast agent, has the potential to enable operator‐independent, time‐efficient, and accurate assessment of cardiac function and aortic anatomy in a single acquisition compared to conventional multi‐slice breath‐hold 2D cine and 3D mDixon MRA at 1.5 T.

## Funding

The authors have nothing to report.

## Conflicts of Interest

Alexandru Cernicanu; Merlijn Sevenster; Jochen Keupp; Jakob Meineke are employees from Royal Philips. Hildo J. Lamb is a consultant for Royal Philips and deputy editor for Cardiovascular Imaging of the *Journal of Magnetic Resonance Imaging*.

## Data Availability

Data generated or analyzed during the study are available from the corresponding author by request.
